# Where Have All the Crop Phenotypes Gone?

**DOI:** 10.1371/journal.pbio.1001595

**Published:** 2013-06-25

**Authors:** Dani Zamir

**Affiliations:** Faculty of Agriculture, The Hebrew University of Jerusalem, Rehovot, Israel

## Abstract

In crop genetics and breeding research, phenotypic data are collected for each plant genotype, often in multiple locations and field conditions, in search of the genomic regions that confer improved traits. But what is happening to all of these phenotypic data? Currently, virtually none of the data generated from the hundreds of phenotypic studies conducted each year are being made publically available as raw data; thus there is little we can learn from past experience when making decisions about how to breed better crops for the future. This ongoing loss of phenotypic information, particularly about crop productivity, must be stopped if we are to meet the considerable challenge of increasing food production sufficiently to meet the needs of a growing world population. Here I present a road map for developing and implementing an information network to share data on crop plant phenotypes.

## The Virtue of Plant Phenotypes

The beauty of life is manifested in phenotypes, the observable characteristics and traits that are produced by an organism's genetic makeup—its so-called genotype. Some phenotypes are caused by single genes, others by multiple genes that can generate different phenotypic outcomes depending on how they interact with each other and with the environment. Phenomics—the systematic study of phenotypes on a genome-wide scale—generates data that are orders of magnitudes more complex to obtain and archive than the four-base nucleic acid code or the twenty amino acids that make up proteins. Unlike the publicly accessible and curated repositories built for the deposition of DNA and protein sequence data, there exists no equivalent public repository for the deposition of raw data generated from the hundreds of plant phenotypic studies conducted each year. This means that data that sometimes costs very large sums of money to generate is lost forever. This lack of phenotype “warehousing," particularly for crop productivity phenotypes, must be stopped if we are to meet the challenge of increasing food production by 70–100% to feed the 9 billion people estimated to populate the earth by 2050 [1].

## Crop Genetics and the Search for Improved Plant Traits

**Figure 1:**
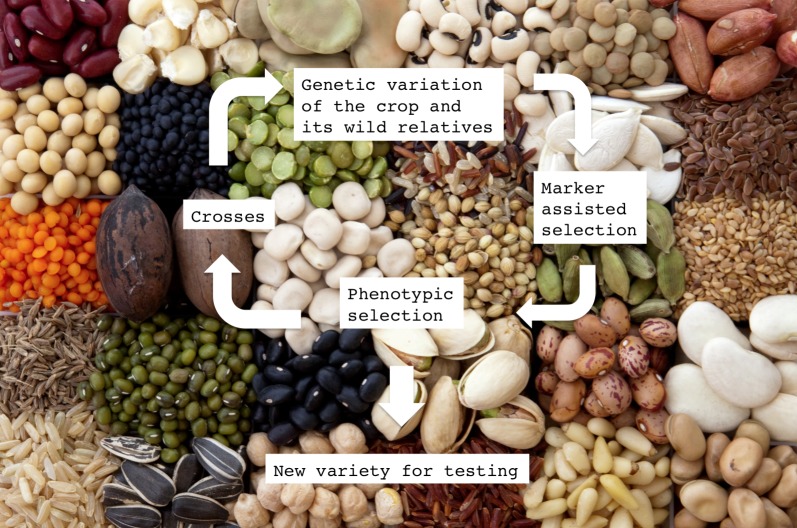
A plant breeding scheme facilitated by the sharing of crop phenotype data. This figure shows the key steps in a plant breeding scheme, which begins with an evaluation of the genetic diversity in a crop and in the wild relatives it can be crossed with. By combining phenotypic selection with marker-assisted selection, the genetic loci (quantitative trait loci, QTL) that associate with certain desirable traits are identified. The “best" individuals are then crossed to create a new gene pool that is enriched for the traits of interest. After several cycles of phenotypic and marker-assisted selection, a plant breeder can then release new crop varieties for testing. The background of the figure shows seeds of different crops that can be measured for homologous phenotypes, such as seed weight, length, width, and the number of seeds produced per plant or per unit area of land (total yield). Such information should be included in shared databases of phenotypes so that it can be linked to syntenic genetic and physical maps in the form of QTL of different crops. This combined information would allow for the definition of new breeding objectives using information gleaned from different crop species. **Image credit**: Naama Rona and Dani Zamir.

Plant breeding is the art and science of improving traits that are of agricultural importance, such as disease resistance or the ability to produce high yields when grown in particular environmental conditions, such as drought (**Figure 1**). Today, the use of thousands of genetic markers to identify the chromosomal regions that are associated with valuable traits increases the speed with which traits can be discovered, verified, and combined in breeding programs [2],[3]. Crop geneticists have analysed over the past decades numerous segregating plant populations in which genomic regions, called quantitative trait loci (QTL; see Glossary, **Box 1**), exist that are associated with agricultural yield. More recently, genome-wide association studies (GWAS) and genomic selection experimental schemes have enriched the repertoire of breeding methods that can be used for finding improved plant traits. Phenotyping for yield and its components, whichever population structure is being used, is a rate-limiting activity of the breeding process since it requires the testing of the genotypes in different years and field environments in order to identify those QTL that more consistently improve the phenotype. Thus it seems incomprehensible that we let such crop genetic studies be published without the deposition of the raw data in appropriate and publicly accessible databases. To give some idea of the scale of the problem, I recently searched the Web of Knowledge ISI database and found over 5,000 publications that report on QTL mapping; for less than 1% of these papers, the raw data is publicly available.

**Box 1. Glossary**

Yield stability: how stable the yield of plant variety is over time and in different cultivation environments.

Quantitative trait: a trait that is influenced by multiple genes and the environment.

DNA marker: a DNA sequence with a known chromosomal location that can be used to identify traits in individuals or populations.

Quantitative trait loci (QTL): chromosomal segments that are closely linked to the genes that underlie quantitative traits.

Introgression lines (ILs): nearly isogenic lines, each containing a single genetically defined chromosomal segment derived from a different breed, strain, or species.

Genome-wide association study (GWAS): an experimental and statistical approach in which numerous genome-wide DNA markers are assayed in different individuals to identify those that are associated with a trait.

Genomic selection: a breeding method that uses phenotypes and genome-wide markers of diverse genotypes to increase the accuracy of future predictions of breeding values.

Synteny: markers that occur in the same order on a chromosome in different species.

## Why Bother to Share Crop Phenotypes?

An analysis of global crop production based on statistics from the United Nation's Food and Agriculture Organization (FAO) shows that crop yields increased by 56% between 1965 and 1985, compared to only 28% from 1985 to 2005 [4]. This initial and significant increase in global crop production was achieved because of the “Green Revolution," in which scientific methods and the use of pesticides, fertilizers, irrigation, mechanization, and soil conservation were successfully applied to the breeding of high-yield varieties of grain crops. A new “revolution" is similarly needed today to increase and accelerate crop yields.

Taking the public sharing of genomic data as an example of the whole being more than its parts, I propose that the more phenotypic data we share, the faster we will achieve crop yield improvements. Making historic phenotypic data publicly available would allow plant researchers to share results, to compare their phenotypes, and to analyse those that have been deposited in the past in order to identify new, and sometimes rare, alleles that improve productivity.

For example, although more than a hundred studies have been conducted in rice (*Oryza sativa*) that involve genetic marker analysis of segregating populations of the two rice subspecies *indica* and *japonica*, the raw phenotypic data for these studies are lost. This lack of phenotype sharing is also relevant to more recent studies in which GWAS was performed on hundreds of rice landraces in the search for the genetic basis of agronomic traits, including grain yield and flowering time [5]. One can imagine a situation in which a scientist finds resistance to a rice pathogen in a few of the above accessions and wants to incorporate the trait into a breeding program. It would make sense to introduce the resistance from the most agriculturally adapted accession. However, without the public availability of data on yield phenotypes, such a decision cannot be made, thus greatly delaying progress. Similar examples can be found for other crops; for example, a high-profile study of heterotic traits in maize hybrids [6] failed to disclose the phenotypes of the tested lines. These examples are just the tip of the iceberg of the practice of nondisclosure of phenotypes in crop plant genetics.

What we need is a publicly accessible bioinformatics resource that would allow plant breeders to explore a multitude of experiments in which traits that are of importance to future agricultural developments, such as yield stability, are available for a variety of crops from different environments and climates (**Box 2**). Efforts have been made to develop such a publically accessible database for the tomato, called the Phenom Networks database (http://phnserver.phenome-networks.com/), in which historical data can be analysed “on the fly." Over the past two decades, our lab has developed and tested tomato interspecific introgression lines (ILs; see Glossary, **Box 1**) consisting of 76 genotypes, each carrying a single “exotic" chromosome segment from a wild species. Phenom Networks harbors raw data from 45 independent IL experiments, in which 355 traits, including yield, morphology, and metabolism, have been measured (the total number of data points currently stands at 443,998). This integrated data management system can go beyond standard QTL identification studies and ventures into a multifaceted systems-level analysis to address questions relating to plant biology [7]. One feature of the Phenom Network platform is its use of a defined ontology—a controlled, standardized vocabulary—to describe phenotypes. Having ontology-defined data for multiple crops on a common computational framework that includes genomes and phenotypes would allow us to revisit the important “law of homologous series in the inheritance and variability" that was articulated by Vavilov in the early 1900s ([8]; **Figure 1**). According to this law, species and genera that are closely related phylogenetically are characterized by having similar potential phenotypic variability. This law implies that knowing the phenotypes of one crop species facilitates their parallel forms to be predicted in other phylogenetically related species. This law reflects a fundamental occurrence in nature, the genetic basis of which can now be investigated mechanistically in regions of synteny (see Glossary, **Box 1**) between related species and the phenomic exercise proposed here.

**Box 2. A data network for crop phenotypes**

In an ideal scenario, phenotypic data could be cross-referenced with, and thus linked to, other plant resources, such as: germplasm resources held in public repositories and gene banks; specific genomic locations on syntenic genetic and physical maps; genes and their expression patterns; and SNPs and their frequencies in association panels. Of key importance is to link phenotypes with the above genomic information (particularly replicated data for complex quantitative phenotypes, such as yield, stress tolerance, etc.), which could be analysed and graphically viewed online or downloaded by anybody for further independent analysis. Users could be allowed to make changes to already existing phenotypic data on a centralized network, such as linking phenotypes to new ontology terms, linking markers to new versions of genomes, correcting mistakes, and so on. The data in this communal system will continuously evolve, rather than being statically stored. Specifically designed algorithms could also be developed to scan the network to compare results between the different trials, organisms, and field conditions to discover new leads.

## How Can We Bring About a Phenotype Sharing Revolution?

I believe plant breeders and geneticists will drive the next agricultural revolution via the web by sharing the phenotypes and genotypes of crop plants using a system that can store, manage, and allow the retrieval of data (**Box 2**). In such a phenome-centric undertaking, the common framework for comparing species will be the description of traits using an ontology and the genetic and physical maps of their respective genomes. The success of such an initiative depends on how effectively phenotypic information gets into the system (**Box 3**). This will depend primarily on the attractiveness and functionality of the tools and the added value they offer to the scientists using them. There is no doubt that having the capacity to compare the results of a specific experiment with those that were conducted in the past would increase the number of degrees of freedom for making discoveries and for more efficiently breeding superior plant varieties.

**Box 3. Phenotypic data: what to deposit and where**

A currently available warehouse for phenotypic data is Dryad (http://datadryad.org/), which is organized by a nonprofit organization and is an international repository for data associated with scientific and medical publications. Data can be downloaded from this site for validation, for further analyses, and for teaching purposes. Presently Dryad houses plant QTL information from 15, mainly non-crop, studies.

As the future development of phenome databases will be assisted by the deposition of experimental data, standards need to be agreed upon for what experimental information would be required. In my view, such data should include:

- Experimental information, including: experimenters' details; publications associated with these data; experimental design; statistical analysis package; field information such as GPS position, planting and harvest dates, soil type, irrigation regimes, and fertilization; and agro-meteorological data.
- Phenotypic information, including: phenotype definition and description in ontological terms; measurements taken; and images of what was measured.
- Genotypic information, including: population structure; method of genotyping; and coordinates of the markers used on genetic and physical maps.
- Curation: other studies conducted on the same population and other studies where the same traits were scored in the same crop.

This endeavour, however, will require the many stakeholders, both private and public, to develop a strong partnership to bring about a change in the culture of phenotype sharing in the plant community. As has been done for genomic data, plant biology funding agencies should require raw data deposition and public availability as a prerequisite for their support of projects. In the meantime, funding organizations worldwide should collaborate to develop shared strategies, concepts, and research money to provide the information technologies required for such an undertaking. Scientific journals, their editors and reviewers, should also require all phenotype and genotype plant breeding data to be deposited and categorized according to an evolving plant ontology [9],[10]. A bioinformatics task for the plant breeding community is to develop web-based resources to display the details of complex phenotypes that will enable hidden biological knowledge to be uncovered from them. The scientific community as a whole ought to start discussing how to link genomes to complex phenotypes across species in a manner that will serve all fields of life science.

What we eat are phenotypes. In view of the global food challenge, it is high time that we find shared ways to link complex phenotypes with plant breeding in changing environments. This urgent need will not cease to exist if ignored; we should start such efforts today.
